# Changes of Physical Function and Quality of Life in Patients with Type 2 Diabetes after Exercise Training in a Municipality or a Hospital Setting

**DOI:** 10.1155/2022/5751891

**Published:** 2022-01-17

**Authors:** Stig Molsted, Trine Munk Jensen, Jane Sedum Larsen, Louise Bockhoff Olesen, Sofie Bjerre Milling Eriksen, Thomas Rehling, Signe Sætre Rasmussen, Mette Aadahl

**Affiliations:** ^1^Department of Clinical Research, Nordsjællands Hospital, Denmark; ^2^Department of Clinical Medicine, Faculty of Health and Medical Sciences, University of Copenhagen, Denmark; ^3^Louiselund, Hørsholm Kommune, Denmark; ^4^Department of Physiotherapy, Nordsjællands Hospital, Denmark; ^5^Department of Endocrinology and Nephrology, Nordsjællands Hospital, Denmark; ^6^Centre for Clinical Research and Prevention, Frederiksberg-Bispebjerg Hospital, Copenhagen, Denmark

## Abstract

**Introduction:**

The aim was to compare changes in physical function and quality of life (QOL) after an exercise training programme to patients with type 2 diabetes mellitus (T2DM) in a municipality and a hospital setting and to compare the patients' physical function and QOL with an age- and sex-matched general population.

**Methods:**

Patients with T2DM were stratified to exercise training in a municipality (*n* = 26) or a hospital (*n* = 46), respectively. The training was one hour twice weekly for 12 weeks. The outcomes were physical function (30 sec chair stand test (CST)) and QOL (using the SF-36). The data for the general population were collected from previous reference studies.

**Results:**

Fifty-one (71%) participants completed the intervention. The CST results improved in both groups with no difference between the municipality and hospital groups (1.6 [0.1; 3.1] *vs.* 3.5 [2.3; 4.8] no., respectively, *p* = 0.062). The QOL scales physical function and general health increased more in the municipality group than in the hospital group (10.5 [2.8; 18.2] *vs.* -1.2 [-7.9; 5.5], respectively, *p* = 0.031, and 8.3 [2.3; 14.4] *vs.* -0.2 [-5.6; 5.1], respectively, *p* = 0.042). Dropout (*n* = 21) during the intervention was associated with reduced QOL at baseline. The patients' CST results at baseline were reduced compared to the general population (11.8 ± 3.5*vs.*18.9 ± 3.3, respectively, *p* < 0.001). All QOL scales apart from social function were reduced in the patients compared to the general population.

**Conclusion:**

Patients in a 12-week exercise training programme in a hospital or a municipality setting had significantly lower QOL compared to an age- and sex-matched population sample. Similar improvements in physical function were observed in patients after completion of the exercise programme irrespective of exercise setting, whereas patient exercising in a municipality setting had higher positive changes in QOL than patients undergoing the same exercise programme in a hospital setting.

## 1. Introduction

Exercise training is a recognized cornerstone in the treatment of hyperglycaemia in type 2 diabetes mellitus (T2DM) [1–5]. Whilst hyperglycaemia is associated with an elevated risk of the development of diabetes complications, other outcomes including physical function and quality of life (QOL) may also have great importance in the patients' daily life. An effect of exercise training on physical function measured using the 30 sec chair stand test (CST) has been suggested [6], whereas the effect on QOL is inconsistent [7–9].

In Denmark, patients with T2DM are referred to rehabilitation programmes including supervised exercise training programmes free of charge [4]. Patients with a relatively low risk of complications are treated in general practice and receive rehabilitation in the municipalities. Patients with a higher risk of diabetes-related complications may be treated and offered rehabilitation in the hospital outpatient clinics or in their municipality [4, 10]. The question remains as to whether patients with T2DM achieve the same positive changes with exercise training, when it is implemented in clinical practice and delivered in a municipality or a hospital setting [11].

The purpose of this study was to compare changes in physical function and QOL after 12 weeks of exercise training twice weekly in a municipality *vs.* a hospital setting in patients with T2DM. In addition, the patients' physical function and QOL were compared with age- and sex-matched data for a general population to investigate the need for rehabilitation regarding these outcomes.

## 2. Materials and Methods

This was a clinical intervention study conducted during the implementation of a training programme for patients with T2DM. The participants were consecutively recruited from Hørsholm municipality and the diabetes outpatient clinic at Nordsjællands Hospital, The Capital Region, Denmark. The patients were referred to exercise training by physicians at the outpatient clinic or by general practitioners in the municipality to the training programme in the hospital and the municipality according to the patients' risk of diabetes complications: the risk assessment was performed on the data for glycaemic control, blood pressure, albuminuria, and micro- and macrovascular complications [4]. The participants were recruited from February 2015 to December 2017, and the study was part of a previous evaluation of changes in musculoskeletal pain with exercise training to the patients [12].

The inclusion criteria were ≥18 years of age, diagnosed with T2DM, and able to perform exercises (could work on an ergometer and walk around in a gym without assistance). The exclusion criteria were participation in a structured exercise training programme ≤ three months of inclusion, dementia, psychiatric disorders, or inability to understand Danish.

The study was approved by the Danish Data Protection Agency and the local scientific ethical committee (ID: H-1-2014-122); it was registered at Clinicaltrials.gov (ID: NTC02366416) and performed in accordance with the Helsinki Declaration. The participants received oral and written information and gave consent.

### 2.1. Outcomes

The participants were tested before and immediately after the 12 weeks of intervention. The outcomes included physical function measured using the 30 sec CST. The test required the participant to rise to a full standing position and return to a seated position as frequent as possible within a 30-second time frame, whilst maintaining their arms folded across their chest [13]. The CST measures physical function and may be used to estimate balance, muscle strength, and power in the lower limbs; QOL was assessed using the self-report questionnaire Short Form 36 (SF-36) [14] with eight subscales rating physical, mental and social health, and well-being, with scores ranging from 0 to 100 where higher scores represented better QOL. Secondary variables included BMI (kg/m^2^) and glycaemic control (HbA_1c_) measured using a Tosoh G8 HPLC Analyzer (Tosoh Bioscience Inc., San Francisco, CA, USA).

Sex, age, employment status, and complications were registered in questionnaires and patient records.

### 2.2. Intervention

The exercise training in the municipality and hospital setting was provided twice weekly for 12 weeks in groups of participants (*n* ≤ 10) and was led by physiotherapists. Each session included one hour of exercises (~10 min warm-up; 15 min interval aerobic exercises on ergometer bikes; 30 min circuit training with aerobic and strength training exercises; and 5 min stretching/cool down). The aerobic exercises should be performed with intensities of 14-16 on the Borg Scale [15]. The strength training was performed with intensities of eight to 12 repetition maximum [16]. In addition, the participants were offered a consultation with a dietician who could give general advice regarding healthy eating to persons with T2DM. Changes in eating habits were not investigated in the study.

### 2.3. Statistical Analyses

The study was performed during the implementation of the exercise training programme and without a fixed number of patients a priori. The number of participants in the study was determined by the number of included patients during the recruitment period.

A chi^2^ test and Welch's *t*-test were used to test for differences between municipality and hospital patient groups at baseline. Differences between the participants' and the general population's CST [17] and SF-36 scores [14] were tested using Student's unpaired *t*-test. The Danish general populations' data for CST (*n* = 1305) [17] and SF-36 (*n* = 4080) [14] were matched with the patients' distributions of age and sex as weights.

To investigate changes in outcomes during the intervention period in all patients, Student's paired *t*-tests were used. The mean between-group differences (municipality *vs.* hospital) in outcome variables were tested using a multiple linear regression analysis. The analyses were adjusted for age, sex, and baseline values of the tested outcomes. The baseline values were included as covariates as they were anticipated to vary between sites. The assumptions of linearity, variance homogeneity, and normal data distributions were assessed by visual inspection of the residuals.

Chi^2^ tests and Welch's *t*-test were used to test for significant differences between the participants who completed the intervention and those who dropped out. Fisher's exact test and Welch's *t*-test were used to test for significant differences between the participants in the municipality and hospital groups who dropped out.

Data are presented as number and percentages, mean ± standard deviation (SD), or mean [95% confidence interval (CI)]. *p* values below 5% were considered statistically significant. The statistical analyses were performed using the IBM SPSS 25 programme.

## 3. Results

123 patients were screened for inclusion, 51 patients declined participation in the clinical study; thus 72 patients (municipality *n* = 26, hospital *n* = 46) were enrolled in the study. The characteristics of the participants in the two groups are presented in Table 1. The hospital group was younger, had higher HbA_1c_ values, had lower CST physical function, and more patients had nephropathy and neuropathy compared to the municipality group. Diabetes duration was 6.3 ± 7.1 years in the municipality group (reported in 26 cases) and 11.8 ± 10.7 years in the hospital group (reported in 15 cases). A total of 51 participants (71%) completed the intervention (municipality group 84%, hospital group 65%). The reasons for dropout were “other disease” (*n* = 9), “time” (*n* = 3), “distance to exercise centre” (*n* = 1), “personal reason” (*n* = 1), and unknown (*n* = 7).

The completers participated in 91% ± 9 of the exercise sessions in the municipality group and 88% ± 12 in the hospital group. No serious adverse events were reported. Missing data at baseline included three missing SF-36 questionnaires.

The participants' baseline physical function measured using the CST and QOL SF-36 scores, except for social function, were significantly reduced compared to age- and sex-matched data for the general population (see Table 2). The Physical Component Scale and the Mental Component Scale scores were also reduced in the patients compared to the Danish general population (39.4 ± 10.2*vs.*48.4 ± 9.1, *p* < 0.001, and 51.0 ± 11.6*vs.*54.8 ± 1.5, *p* = 0.012, respectively).

In all participants, the CST, role limitation physical, vitality, and role limitation emotional increased from baseline to follow-up after the intervention period (see Table 3). The patients' Physical Component Scale increased from 41.2 ± 9.2 to 43.9 ± 10.6, *p* = 0.018, whereas the Mental Component Scale remained unchanged (from 54.0 ± 10.6 to 55.9 ± 9.4, *p* = 0.189).

In the adjusted complete case analyses, the CST results increased in the municipality and hospital groups without difference between the sites (Table 4). The physical function and general health scores of the SF-36 increased in the municipality group with greater changes in the municipality group. The BMI decreased more in the municipality group compared to the change in the hospital group (-1.2 [-1.8; -0.5] *vs.* -0.2 [-0.7; 0.4] kg/m^2^, respectively, *p* = 0.026). There was no difference in change of HbA_1c_ between the municipality and hospital group (-7.8 [-11.5; -4.1] *vs.* -4.5 [-7.7; -1.2] mmol/mol, respectively, *p* = 0.217).

A total of 21 participants (29%) dropped out of the intervention (municipality group *n* = 5 (19%), hospital group *n* = 16 (35%)). At baseline, the participants who dropped out scored significantly lower on all subscales of the SF-36 (Table 5). Likewise, there were differences between the dropouts in the municipality group and the hospital group at baseline in the variables age (68 ± 5*vs.*59 ± 12 years, *p* = 0.023), HbA_1c_ (50 ± 15*vs.*73 ± 19 mmol/mol, *p* = 0.024), BMI (31.2 ± 4.2*vs.*36.8 ± 5.4 kg/m^2^, *p* = 0.042), and CST (14.0 ± 3.2*vs.*9.8 ± 3.1 no., *p* = 0.035), respectively.

## 4. Discussion

The main result of this study was that exercise training in patients with T2DM was associated with similar positive changes in physical function whether performed in a municipality or a hospital setting, whereas the positive changes in QOL were more pronounced in the municipality group. Physical function and QOL were reduced in the included patient sample compared to the general population prior to the intervention, which emphasize the need for exercise training in this group of patients.

Whilst there were no differences in the positive changes in the CST between the two groups with the training, the QOL scores in physical function and general health increased in the municipality group only. The reason for the less positive change in QOL in the hospital setting may be associated with differences between the groups at baseline. Although baseline outcome values were adjusted for in the statistical analyses, the hospital group was characterized by a more severe diabetes status with an impaired glycaemic control and more frequent complications including neuropathy and nephropathy compared to the municipality group. A more severe disease status may counteract positive changes with exercise training on QOL and is therefore a likely explanation to the difference in changes.

The study also showed a significantly reduced physical function measured using the CST and QOL in the patients at baseline compared to the general population. This was true even though the patients were a selected sample in terms of patients that accepted invitation to participate in an exercise training programme. This demonstrates a crucial need for rehabilitation programmes for these patients. Not only were SF-36 dimensions physical function and role limitation physical reduced in the patients, vitality was also seriously reduced in patients compared to the population sample. A reduced vitality measured using the SF-36 may be a result of fatigue that is a well-known symptom in many patients with chronic diseases. As anticipated, bodily pain was also more pronounced in the patients than in the population sample, as reported in other studies on T2DM [12, 18].

It was not anticipated that the self-rated physical function and general health remained unchanged across all patients after the exercise programme. However, the unchanged score in physical function may most likely be a result of the differences between the groups with a more pronounced positive change in the municipality group compared to the hospital group as discussed above. The inconsistent changes of QOL have been presented in other trials. Two previous RCTs suggested positive effects of exercise training on QOL in patients with T2DM without severe diabetes complication [7, 8]. However, the evidence of the association between exercise training and QOL in patients with T2DM is inconstant [9].

The study suffered from reduced adherence as more than a fourth of the participants dropped out of the study. The dropout rate was the highest in the hospital group, which may be the result of a patient group with a more impaired health status and a longer transportation to the training clinic compared to the municipality group. However, the present dropout rate may be anticipated as the intervention was implemented in a clinical practice with a patient sample recruited with few exclusion criteria. Whilst the dropout rate was relatively high, the attendance in the group of participants who completed the intervention was good (~90%).

The analyses revealed that QOL at baseline was reduced among dropouts compared to the completers, which could indicate that impaired QOL reduces the personal resources and motivation for completing the training programme. Quality of life includes mental health, and a previous study found that symptoms of depressions in patients with T2DM were associated with a 49% increased risk of dropping out of a training programme [19].

This study may have important clinical implications in terms of positive effects of implementation of training on physical function and QOL. Indeed, an improved physical function in daily living may be an important motivational factor for exercise training in patients with T2DM, who have lower levels of physical activity compared to a nondiabetic population [20]. The improved physical function was found even though only two exercise sessions were performed weekly, whilst most other exercise trials have used three exercise sessions weekly to show positive effects on different outcomes [1–3].

An important limitation of the study was the relatively low number of participants. With the clinical practice design of the present study, it is unknown whether patients in the municipality setting group would have achieved greater positive changes in the hospital setting and *vice versa*. However, the scope of the study was not to test the effect of the two different exercise settings, but rather to evaluate changes in two different sites in clinical practice. In addition, data for usage of glucose-lowering medication and eating habits were unfortunately not obtained, and such cointerventions could have affected some of the tested outcomes.

In conclusion, there was no difference between the positive changes after implemented exercise training in clinical practice on objectively measured physical function in patients with T2DM treated in a municipality and a hospital. Changes in QOL with exercise training were more pronounced in the municipality group. The included patients had reduced physical function and QOL at baseline compared to an age- and sex-matched general population; thus, the need for rehabilitation to this patient group is crucial. Dropout from the exercise training programme was associated with reduced QOL prior to the intervention. Future strategies to improve the benefits of the exercise training interventions to patients with T2DM should have a focus on participation and prevention of dropout in mentally and physically frail patients.

## Figures and Tables

**Table 1:** Baseline characteristics of 72 patients with T2DM stratified by rehabilitation sites.

Variables	Municipality (*n* = 26)	Hospital (*n* = 46)	*p*
Sex (m/f)	12/14	27/19	0.305
Age (years)	69.8 ± 10.7	62.6 ± 9.5	0.005
BMI (kg/m^2^)	32.6 ± 6.4	35.5 ± 5.0	0.054
Employed (yes/no)	1/25	13/31	0.009
Chair stand test (no.)	13.2 ± 3.6	11.0 ± 3.2	0.013
Complications			
Nephropathy	2 (7.7%)	15 (32.6%)	0.017
Arthritis	9 (34.6%)	12 (26.1%)	0.444
Neuropathy	4 (15.4%)	22 (47.8%)	0.006
Cardiovascular disease	9 (34.6%)	22 (47.8%)	0.227
HbA_1c_ (mmol/mol)	50.9 ± 10.1	69.4 ± 16.2	<0.001
SF-36 (0-100)			
Physical function	59.3 ± 27.1	60.5 ± 23.8	0.854
Role physical	46.0 ± 39.3	45.9 ± 42.6	0.995
Bodily pain	53.6 ± 26.9	57.7 ± 28.0	0.555
General health	60.5 ± 17.8	57.9 ± 22.1	0.597
Vitality	53.3 ± 22.6	52.7 ± 27.0	0.922
Social function	84.6 ± 21.0	85.8 ± 24.3	0.838
Role emotional	50.7 ± 42.1	61.1 ± 45.3	0.344
Mental health	74.5 ± 20.3	74.7 ± 20.7	0.963

Data are presented as the mean ± SD or as *n* (%).

**Table 2:** Physical function and QOL in the included patients with T2DM and in an age- and sex-matched general population.

Variables	Patients with T2DM (*n* = 72)	Danish general population	*p*
Chair stand test (no.)	11.8 ± 3.5	18.9 ± 3.3	<0.001
SF-36 (0-100)			
Physical function	60.0 ± 24.9	76.3 ± 9.3	<0.001
Role physical	46.0 ± 41.1	67.9 ± 11.0	<0.001
Bodily pain	56.1 ± 27.4	76.7 ± 4.2	<0.001
General health	58.9 ± 20.5	67.5 ± 4.7	0.001
Vitality	52.9 ± 25.3	67.9 ± 4.7	<0.001
Social function	85.3 ± 23.0	88.9 ± 3.9	0.213
Role emotional	57.2 ± 44.1	79.2 ± 7.7	<0.001
Mental health	74.6 ± 20.4	81.7 ± 2.8	0.005

Data are presented as the mean ± SD.

**Table 3:** Baseline and postintervention results of physical function and QOL in patients who completed the 12-week exercise training intervention.

Variables	Baseline	Postintervention	*p*
Chair stand test (no.)	12.2 ± 3.4	14.9 ± 4.2	<0.001
SF-36 (0-100)			
Physical function	64.2 ± 22.4	68.3 ± 25.7	0.113
Role physical	52.6 ± 41.5	72.4 ± 36.5	0.001
Bodily pain	59.2 ± 27.2	64.9 ± 27.7	0.071
General health	63.5 ± 19.4	67.2 ± 19.4	0.081
Vitality	57.9 ± 24.9	65.1 ± 23.1	0.002
Social function	91.2 ± 15.7	89.0 ± 17.6	0.253
Role emotional	66.0 ± 41.4	82.6 ± 28.9	0.002
Mental health	79.4 ± 19.0	82.4 ± 16.1	0.154

Data are presented as the mean ± SD.

**Table 4:** Changes of physical function and QOL from baseline to postintervention by intervention groups. Complete case analyses adjusted for age, sex, and the outcome variable's baseline value.

	Municipality (*n* = 21)	Hospital (*n* = 30)		
Variables	Change in group	Change in group	Between groups	*p*
*Δ* CST (no.)	1.6 [0.1; 3.1]	3.5 [2.3; 4.8]	1.9 [-0.1; 3.9]	0.062
SF-36 (0-100)				
*Δ* physical function^c^	10.5 [2.8; 18.2]	-1.2 [-7.9; 5.5]	-11.7 [-22.2; -1.1]	0.031
*Δ* role physical^c,d^	20.6 [5.7; 35.5]	18.2 [5.6; 30.7]	-2.5 [-22.5; 17.6]	0.807
*Δ* bodily pain^b^	4.9 [-4.4; 14.2]	7.4 [-0.7; 15.5]	2.5 [-10.4; 15.3]	0.699
*Δ* general health^c^	8.3 [2.3; 14.4]	-0.2 [-5.6; 5.1]	-8.6 [-16.9; -0.3]	0.042
*Δ* vitality^c^	9.5 [3.1; 16.0]	5.7 [-0.1; 11.4]	-3.8 [-12.7; 5.0]	0.388
*Δ* social function^c^	-2.6 [-8.6; 3.4]	-1.8 [-7.1; 3.5]	0.8 [-7.4; 9.0]	0.846
*Δ* role emotional^b^	20.3 [8.5; 32.1]	14.2 [4.5; 24.0]	-6.1 [-21.9; 9.8]	0.444
*Δ* mental health^c^	0.9 [-4.4; 6.2]	4.2 [-0.4; 8.8]	3.2 [-3.9; 10.4]	0.368

Data are presented as mean changes [95% CI]. CST: 30 sec chair stand test. *N* varies in the municipality group: ^d^*n* = 20. *N* in the hospital group: ^a^*n* = 27, ^b^*n* = 28, and ^c^*n* = 29.

**Table 5:** Baseline characteristics of completers *vs*. dropouts.

Variables	Completers (*n* = 51)	Dropouts (*n* = 21)	*p*
Sex (m/f)	29/22	10/11	0.474
Age (years)	66.7 ± 9.8	61.4 ± 11.2	0.059
HbA_1c_ (mmol/mol)^bc^	60.6 ± 14.8	67.2 ± 2.8	0.222
Weight (kg)	100.5 ± 19.2	106.3 ± 18.4	0.236
BMI (kg/m^2^)	34.0 ± 5.7	35.4 ± 5.6	0.328
Employed	9 (17.6%)	5 (23.8%)	0.420
Complications			
Nephropathy	13 (25.5%)	4 (19.0%)	0.558
Arthritis	13 (25.5%)	8 (38.1%)	0.285
Neuropathy	16 (31.4%)	10 (47.6%)	0.192
Cardiovascular disease	24 (47.0%)	7 (33.3%)	0.285
CST (no.)	12.2 ± 3.4	10.8 ± 3.6	0.135
SF-36 (0-100)			
Physical function^bc^	64.2 ± 22.4	48.9 ± 28.3	0.043
Role physical^bc^	52.6 ± 41.5	28.9 ± 35.6	0.025
Bodily pain^bc^	60.3 ± 27.1	44.8 ± 25.8	0.040
General health^fc^	63.4 ± 19.6	46.9 ± 18.1	0.002
Vitality^bc^	58.6 ± 24.7	37.5 ± 20.8	0.001
Social function^bc^	91.0 ± 15.8	70.4 ± 31.5	0.013
Role emotional^acd^	66.7 ± 40.8	31.5 ± 43.5	0.006
Mental health^bc^	79.4 ± 19.0	62.1 ± 19.0	0.002

Data are presented as the mean ± SD or *n* (%). CST: 30 sec chair stand test. Completers of the municipality group: ^a^*n* = 4. Completers of the hospital group: ^b^*n* = 29 and ^d^*n* = 28. Dropouts of the hospital group: ^c^*n* = 14.

## Data Availability

The raw data are not available according to the rules of the Danish Data Protection Agency.
